# Comparative cross-sectional study of vegan and omnivorous diets and their impact on cardiac function among endurance athletes

**DOI:** 10.6026/973206300214350

**Published:** 2025-12-15

**Authors:** Glenn Jo, Shubhi Tamrakar, Sorabh Sharma, Shanmukha Koppolu, E. Emi Goldy, Sailesh I.S Kumar

**Affiliations:** 1 Department of Internal Medicine, Kerala State Medical Council, Kerala, India; 2 Department of Physiology, MGM Medical College Panvel, Navi Mumbai, Maharashtra, India; 3 Department of Medicine, University of Arizona, Arizona, USA; 4 Department of Trauma and Orthopaedics, Barts NHS Trust, London, England, United Kingdom; 5 Department of Biochemistry, Panimalar Medical College Hospital and Research Institute Varadharajapuram, Poonamallee, Chennai, Tamil Nadu, India; 6 Department of Surgery, Madras Medical College, Chennai, Tamil Nadu, India

**Keywords:** Vegan diet, omnivorous diet, endurance athletes, cardiac function, echocardiography, VO_2_ max

## Abstract

Vegan and omnivorous diets are both common in endurance sport; however, the chronic effects of these diets on cardiac function remain uncertain. Therefore, it is of interest to compare echocardiographic, vascular and exercise performance between endurance athletes on vegan or omnivorous diets. Data shows there is comparable systolic function; however, vegans displayed superior diastolic function and arterial compliance; whilst omnivores maintained higher hemoglobin and ferritin levels. Thus, we show that diet can influence distinct aspects of cardiovascular adaptation without limiting endurance capacity.

## Background:

The term "vegan diet" comprises a range of eating patterns that prioritize nutrient-rich foods such as fruits and vegetables, legumes, whole grains, nuts and seeds. In comparison to omnivorous diets, which are often lower in such products, the vegan diet has been favourably connected with changes in cardiovascular disease (CVD) risk markers such as reduced body mass index (BMI) values, total serum cholesterol, serum glucose, inflammation, blood pressure and day to day nutritional requirements [1]. Nutrition is a key element of cardiovascular health and when we consider the role of nutrition in endurance athletes, there are unique aspects of nutrition and physical training that together promote physiological adaptation. Endurance performance requires on-going challenge to the cardiovascular system, with direct adaptations of both structure and function that have collectively been described as an "athlete's heart". Physiological changes include increases in left ventricular (LV) mass, stroke volume and chamber dimensions of the heart, all of which serve to increase cardiac output and consequently, aerobic exercise performance [2]. Typically, these changes, while considerable, are not regarded as pathological, yet little is known about the habits of eating over time and how nutrition impacts such remodeling. Because a vegan diet (there are competing definitions of "vegan") is characterized as involving no animal-based food products, it has become popular and it is becoming recognized by health professionals [3]. There is also not emerging but consistent literature demonstrating lower cardiovascular risk metrics (lipids profile, blood pressure, vascular function being the most studied) and lower risk of ischemic heart disease with vegan dietary habitual eating [4]. A vegan diet accounts for a relatively high total food mass and offers ample opportunity for fruits and vegetables, but a vegan diet also often comes with long-term low or limited intake of certain types of foods that may confer risk on cardiovascular health- this may include plant range diversity, antioxidant levels, phytochemical intake, plant fiber intake and others [5]. Further, such strong restriction can dramatically restrict the intake of foods determined beneficial animal food products with high biological value protein sources, bioavailable vitamin B12 and heme iron. Understanding how to get these compounds in one's diet, along with key nutrients for hemoglobin synthesis, oxygen transport and muscle recovery seems crucial [6]. Conversely, omnivorous diets allow for consumption of higher quality animal proteins; heme iron; and long-chain omega-3 fatty acids that promote erythropoiesis, hemoglobin concentration and recovery following exercise. Previous reports have indicated that omnivorous endurance athletes frequently have higher hemoglobin indices and higher maximal oxygen uptake (VO_2_ max), alluding to improved oxygen delivery and utilization during extended exercise [7]. However, it is conceivable that such improvements could be counterbalanced with fairly poor vascular profiles, relative to blacks' diets, as omnivorous dietary patterns are frequently associated with higher intake of saturated fats (i.e. greater levels of saturated fats); lowering arterial compliance; increasing cardiovascular risk chronically [8]. Cardiovascular outcomes of dietary patterns may be of great concern to endurance athletes, as modest changes in diastolic relaxation, ventricular filling, or systemic vascular resistance would likely lead to affect exercise tolerances or cardiac remodel over the longer term. In this regard, it is worth noting that echocardiography can non-invasively assess quantities such as left ventricular ejection fraction, diastolic function indices (E/A- ratio, E/e') and left atrial volume index; cardiopulmonary exercise testing non-invasively measures aerobic capacity (functional VO_2_ max); and measures of arterial stiffness, such as pulse wave velocity can quantify vascular adaptations that may be dependent on a dietary pattern [9]. These assessments collectively provide an overview of how diet, in conjunction with training, affects cardiovascular function in athletes. Therefore, it is of interest to compare the effects of vegan versus omnivorous diets on cardiac remodeling, vascular health and exercise performance in endurance athletes.

## Materials and Methods:

The present study was an observational cross-sectional study in which 80 competitive endurance athletes (40 vegan and 40 omnivore) were enrolled. Participants were aged between 18 and 35 years and had at least three years of competition experience. Athletes were recruited through existing local sports and university athletic club programs and training facilities. Dietary status was confirmed at the time of enrollment using a food frequency questionnaire that has been previously validated, confirming that participants had followed either a vegan diet or an omnivorous diet for a minimum of two years prior to study enrollment. Exclusion criteria consisted of a self-reported history of cardiovascular disease, diabetes mellitus, smoker status, or use of performance-enhancing drugs, to minimize confounding factors impacting cardiac outcomes. Clinical evaluation of participants occurred following enrollment into the study. Each echocardiographic evaluation was conducted by a qualified cardiologist using standard two-dimensional and Doppler imaging at rest according to applicable guidelines set forth by the American Society of Echocardiography. Left ventricular ejection fraction (LVEF), left atrial volume index (LAVI) and various parameters related to diastolic function (E/A ratio, E/e' ratio) were measured. Non-invasive measurements of arterial function were recorded using pulse wave velocity, a well-validated measure of arterial stiffness and vascular compliance. To evaluate exercise capacity, all athletes were administered a graded treadmill exercise test with standardization and respiratory gas exchange was evaluated to determine maximal oxygen uptake (VO_2_ max). Tests were given under medical supervision; each athlete was instructed not to perform any moderate to intense training for the 24 h preceding testing so that the testing would be conducted under similar conditions. In addition, venous blood samples were taken following an overnight fast for hematological assessments of hemoglobin concentration, hematocrit and serum ferritin levels. These parameters were indicative of oxygen carrying capacity and iron storage status and were influenced by dietary intake. Demographic and training-related data on baseline information (age, sex, training duration, weekly training hours) was obtained from structured interviews and verified wherever possible with competition records (athletic history). These data were used to enable comparisons of group characteristics and verified that both groups were similarly matched in their training exposure and athletic background. Statistical analyses were performed using SPSS software. Continuous variables were reported as mean ± standard deviation and compared between groups using the student's t-test and categorical variables were reported using the chi-square test. A p-value of less than .05 was used to indicate statistical significance. The study protocol was reviewed and approved by the institutional ethics committee and written informed consent was obtained from all participants prior to data collection.

## Results:

In total, 80 endurance athletes (40 vegans; 40 omnivores) were included in the analysis. The two groups were well matched on age, sex, training duration and weekly training hours to limit baseline confounding. The echocardiographic analysis revealed appropriate left ventricular ejection fraction and left atrial volume index measures between groups, while vegan athletes exhibited better diastolic function. Cardiopulmonary exercise testing demonstrated a non-significant trend for omnivores to have a higher VO_2_ max, while the hematological tests demonstrated that omnivores had significantly higher hemoglobin and ferritin concentrations; arterial function testing demonstrated that the vegan athletes had better vascular compliance, as indicated by lower pulse wave velocity. Overall, these findings suggest that both vegan and omnivorous diets can support adaptations to endurance training, but have unique influences on cardiac, hematological and vascular measures. Table 1 (see PDF) confirmed that baseline demographic and training variables were comparable between groups. Table 2 (see PDF) showed no differences in systolic function but demonstrated significantly better diastolic function in vegan athletes. Table 3 (see PDF) highlighted the hematological advantage of omnivores, with higher hemoglobin and ferritin values, though VO_2_ max differences were not significant. Table 4 (see PDF) revealed superior arterial compliance in vegan athletes, as evidenced by lower pulse wave velocity. Together, the results indicate distinct but complementary adaptations in endurance athletes depending on dietary pattern.

## Discussion:

This study compared the effects of vegan and omnivorous diets on cardiac function, vascular health and exercise performance in endurance athletes. Both dietary groups demonstrated preserved systolic function, indicating that high-level endurance performance is compatible with either diet. However, differences were observed in diastolic parameters, hematological measures and vascular function, suggesting diet-specific adaptations. Vegan athletes exhibited better diastolic function and superior arterial compliance, whereas omnivorous athletes showed higher hemoglobin and ferritin concentrations, which may contribute to enhanced oxygen transport. These findings emphasize that while both diets support endurance training, they promote distinct physiological profiles with potential long-term implications for cardiovascular health [9]. The observation of comparable left ventricular ejection fraction in both groups indicates that dietary pattern does not impair systolic performance, consistent with previous literature reporting preserved ventricular contractility in endurance-trained individuals regardless of diet [10]. However, vegan athletes demonstrated a higher E/A ratio and lower E/e' ratio, reflecting more favorable diastolic relaxation and myocardial compliance. These findings align with earlier reports linking plant-based nutrition to improved endothelial function and reduced vascular stiffness [11]. In comparison to omnivorous athletes, vegan athletes showed enhanced vascular compliance, based on lower measures of pulse wave velocity which additionally support the vascular protective and cardioprotective roles of plant-based diets for long-term cardiovascular health [12]. Omnivorous athletes, on the other hand, had higher hemoglobin and ferritin values, which correspond to the greater bioavailability of heme iron in animal-based foods. These factors are clinically relevant, given that hemoglobin and ferritin are directly related to oxygen-carrying capacity and likely account for the non-significant trend to higher VO_2_ max in the omnivorous group. While endurance athletes consuming animal products generally demonstrate better iron status and greater aerobic capacity, iron deficiency is a risk for a vegan athlete. Previous literature suggests that, with thoughtfully planned dietary menus and supplementation, vegan athletes can still have adequate iron stores [13]. The study's results substantiate both dietary patterns provide complementary benefits: a vegan diet appears advantageous for vascular and diastolic function, while an omnivorous diet supports hematological reserves and oxygen delivery. From a performance perspective, the non-significant differences in VO_2_ max illustrate that both dietary patterns can support the function of an individual's endurance and aerobiology provided nutritional adequacy. The main takeaway from the results is the importance of establishing individualized dietary patterns that optimize both cardiovascular benefits and hematological tracking, especially considering the demands of endurance athletes [14]. The strengths of this study are strong, including matching age, training history and competition level between vegan and omnivorous athletes, employing standardized echocardiography, vascular measures and cardiopulmonary exercise testing to assess performance potential; however, limitations must be noted. The design of the study was cross-sectional and does not allow for cause and effect to be delivered regarding diet and adaptation of cardiac function. Dietary intake was active and therefore may be susceptible to recall error. The current sample did find some differences in some of the outcomes that we examined with athletes, which scaffolds the suggestion for further investigation into more widespread trends across athletic populations, although more research is needed in more diverse athletic populations [15]. In addition, the long-term outcomes related to cardiac remodeling based on dietary patterns are warranted with longitudinal studies. The evidence of the current study indicates that both vegan and omnivorous nutrition supports endurance training but yields different cardiovascular adaptations. Specifically vegan athletes were observed to develop enhanced diastolic function and vascular compliance, while omnivorous athletes did have elevated hemoglobin and ferritin levels contributing to greater oxygen carrying capacity indicative of training adaptations. The findings affirm the necessity conducting individual nutrition plan for endurance sports; specifically, for vegan athletes to monitor iron status and for omnivorous athletes to continue monitoring vascular health. Furthermore, longitudinal studies are needed to evaluate whether these adaptations attenuate cardiovascular benefits in the future and evaluation of performance.

## Conclusion:

Both vegan and omnivorous diets support endurance performance but result in distinct cardiovascular adaptations, with vegans showing superior diastolic and vascular function while omnivores maintain stronger hematological reserves. Thus, we show the importance of individualized nutrition planning to optimize both heart health and athletic performance in endurance sports.
